# Microglia in Health and Disease: The Strength to Be Diverse and Reactive

**DOI:** 10.3389/fncel.2021.660523

**Published:** 2021-03-31

**Authors:** Oihane Uriarte Huarte, Lorraine Richart, Michel Mittelbronn, Alessandro Michelucci

**Affiliations:** ^1^Luxembourg Centre for Systems Biomedicine (LCSB), University of Luxembourg, Esch-sur-Alzette, Luxembourg; ^2^Luxembourg Center of Neuropathology, Luxembourg, Luxembourg; ^3^Department of Oncology (DONC), Luxembourg Institute of Health (LIH), Luxembourg, Luxembourg; ^4^Faculty of Science, Technology and Medicine, University of Luxembourg, Belvaux, Luxembourg; ^5^National Center of Pathology (NCP), Laboratoire National de Santé (LNS), Dudelange, Luxembourg; ^6^Neuro-Immunology Group, Department of Oncology (DONC), Luxembourg Institute of Health (LIH), Luxembourg, Luxembourg

**Keywords:** microglia, heterogeneity, brain regions, neuroinflammation, neurodegenerative diseases, brain tumors

## Abstract

Microglia are the resident immune effector cells of the central nervous system (CNS) rapidly reacting to any perturbation in order to maintain CNS homeostasis. Although their outstanding reactive properties have been elucidated over the last decades, their heterogeneity in healthy tissue, such as across brain regions, as well as their diversity in the development and progression of brain diseases, are currently opening new avenues to understand the cellular and functional states of microglia subsets in a context-dependent manner. Here, we review the main breakthrough studies that helped in elucidating microglia heterogeneity in the healthy and diseased brain and might pave the way to critical functional screenings of the inferred cellular diversity. We suggest that unraveling the cellular and molecular mechanisms underlying specific functionalities of microglial subpopulations, which may ultimately support or harm the neuronal network in neurodegenerative diseases, or may acquire pro- or anti-tumorigenic phenotypes in brain tumors, will possibly uncover new therapeutic avenues for to date non-curable neurological disorders.

## Introduction

Microglial cells are the innate immune cells of the brain and key players in maintaining the homeostasis of the central nervous system (CNS) (Sierra et al., 2016). Microglia originate from erythro-myeloid precursors in the yolk sac and migrate to the brain around embryonic day 9.5 in mouse (Ginhoux et al., 2010), while they colonize the human cerebrum between the 4th and 24th week of gestation (Menassa and Gomez-Nicola, 2018). Their ontogeny, together with their slow turnover, which differentiate them from most other hematopoietic lineages in adult individuals (Réu et al., 2017), as well as the local environment in the CNS, make microglia a distinct immune cell population (Sousa et al., 2017). Until approximately 20 years ago, microglia have been considered as a resident resting cell type of the healthy CNS able to react to pathogens or toxic elements. However, this paradigm has shifted into the concept of “surveillant” and “supporting” microglia exerting additional multiple functional roles, such as neuromodulation and phagocytosis (Gomez-Nicola and Perry, 2015). For example, during development microglia contribute to building the neuronal circuit through synaptic pruning and stripping, phagocytosis of dying neurons and secretion of neurotrophic factors (Paolicelli et al., 2011; Ekdahl, 2012; Schafer et al., 2012; Paolicelli and Ferretti, 2017; Scott-Hewitt et al., 2020). Further, it has recently been shown that an ATP-dependent microglia-driven negative feedback mechanism operates similarly to inhibitory neurons and is essential for protecting the brain from an excessive activation (Badimon et al., 2020). Taken together, due to their multiple critical functional roles in the homeostatic brain, various neurological disorders, including neurodegenerative diseases and brain tumors, implicate microglia. Briefly, in Parkinson’s disease (PD) and Alzheimer’s disease (AD), a mix of beneficial and detrimental roles of microglia have been suggested (Wyss-Coray and Mucke, 2002; Glass et al., 2010; Bodea et al., 2014; Joers et al., 2017; Salter and Stevens, 2017; Wolf et al., 2017; Duffy et al., 2018). For example, in AD microglial cells have been associated with the phagocytosis and degradation of amyloid-ß plaques, but the subsequent excessive release of cytokines is supposed to contribute to neuronal loss (Salter and Stevens, 2017). Similarly, in PD, where microglia are able to recognize and engulf alpha-synuclein, but the concomitant release of reactive oxygen species (ROS) or pro-inflammatory mediators can actively contribute to neurodegeneration (Glass et al., 2010). In brain tumors, microglia, along with tumor-infiltrating macrophages, constitute the predominant immunological cell types (Graeber et al., 2002) and have been shown to affect tumor progression as well as patient survival (Morimura et al., 1990; Gieryng et al., 2017; Sorensen et al., 2018). Indeed, tumor-associated microglia/macrophages (TAMs) are key players along tumor development by contributing to the establishment of a tumor-supporting microenvironment (Hambardzumyan et al., 2016; Grabowski et al., 2020; Maas et al., 2020).

In this context, the hypothesis that several microglial cell subsets exist in the brain has gain momentum in the recent years and microglial heterogeneity has been addressed from different points of views, including morphology, cellular density, proliferation capacity as well as transcriptional and proteomic signatures (De Biase and Bonci, 2018; Silvin and Ginhoux, 2018). Additionally, significant advances have been made by taking advantage of the recently developed single-cell technologies, including RNA-sequencing and mass cytometry (CyTOF). Indeed, several studies using these approaches have now confirmed that microglial cells represent a complex population constituted by different subsets, both in the healthy and diseased brain, displaying specific neuroimmunological adaptations in a context-dependent manner (Stratoulias et al., 2019; Masuda et al., 2020; Provenzano et al., 2020).

Here, we will review the main breakthrough studies that helped to elucidate microglia heterogeneity in the healthy and diseased brain. We suggest that unraveling the cellular and molecular mechanisms underlying specific microglia subpopulations might contribute to uncover new therapeutic targets for brain disorders with an immunological component, including neurodegenerative diseases and tumors.

## Microglia Heterogeneity in the Healthy Brain

**FIGURE 1:**
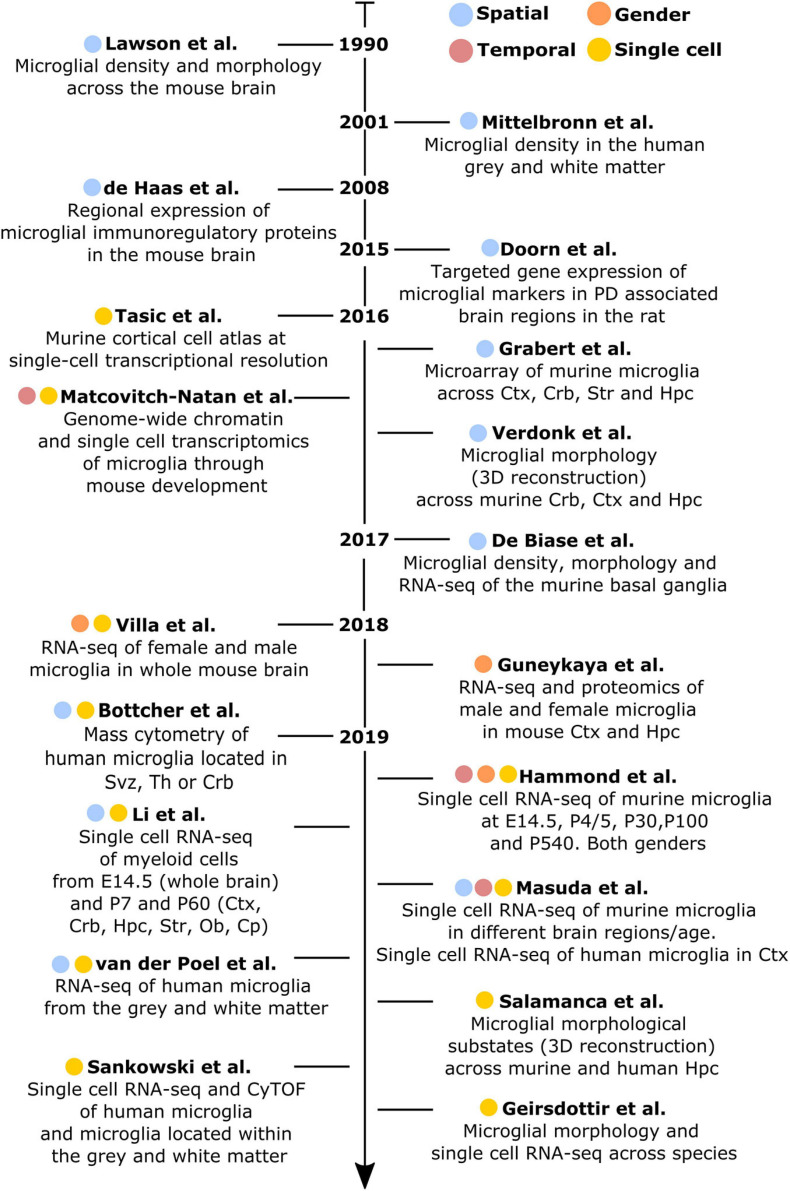
Chronologically ordered list of the main studies that have contributed to elucidate microglial diversity in the healthy brain. Studies are color-coded based on their corresponding addressed topics: spatial (blue), temporal (red), gender (orange), or single-cell resolution (yellow). Ctx, cortex; Crb, cerebellum; Str, striatum; Hpc, hippocampus; Svz, subventricular zone; Th, thalamus; Ob, olfactory bulb; Cp, corpus callosum.

**FIGURE 2:**
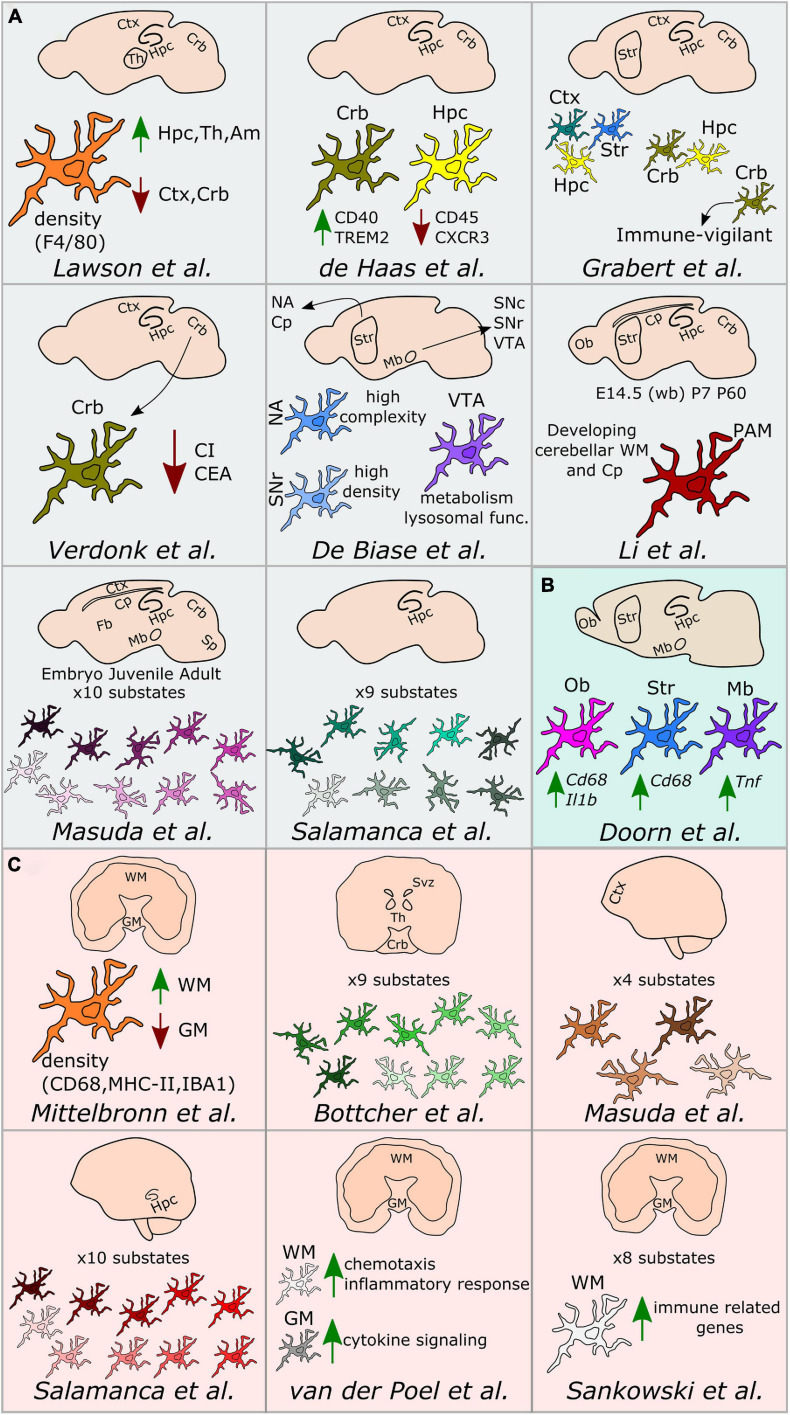
Regional microglial heterogeneity in the healthy brain. Schematic representation of the main findings over the main studies addressing microglial heterogeneity across mouse **(A)**, rat **(B)**, and human **(C)** brain regions. Ctx, cortex; Crb, cerebellum; Str, striatum; Hpc, hippocampus; Svz, subventricular zone; Th, thalamus; Ob, olfactory bulb; Cp, corpus callosum; Mb, midbrain; NA, nucleus accumbens; VTA, ventral tegmental area; SNc, substantia nigra pars compacta; SNr, substantia nigra pars reticulata; Sp, spinal cord; Fb, forebrain; WM, white matter; GM, gray matter; PAM, proliferative-region-associated microglia; WB, whole brain; CI, complexity index; CEA, covered environment area.

An extensive characterization of microglial heterogeneity encompassing fundamental aspects, including development, gender specificities and spatial distribution has been conducted in the healthy brain. Hence, along this chapter, we chronologically review the main studies that contributed to acquire the current knowledge of microglia diversity under homeostatic conditions (Figures 1, 2).

Lawson et al. (1990) have conducted the first study addressing microglial heterogeneity in the 90’s. The authors showed that, in the mouse brain, microglial ramifications and cell shapes were region-dependent. By using the macrophage marker F4/80, they also reported divergent microglial densities across specific brain regions, with higher density in the hippocampus, thalamus and amygdala compared to the cortex and cerebellum (Lawson et al., 1990). Concomitantly to this study in mice, Mittelbronn et al. (2001) studied microglial density across human brain regions using myeloid-specific-immunological markers, including CD68, MHC-II, and IBA1. In this study, the authors described a higher content of microglial cells within the white-matter when compared to gray-matter (Mittelbronn et al., 2001). Seven years later, de Haas et al. (2008) took advantage of *ex vivo* flow cytometry analyses to investigate regional differences in the expression of immunoregulatory proteins across the mouse spinal cord and various brain regions, such as the cerebral cortex, hippocampus, cerebellum or striatum. Interestingly, the authors described that CD40 was overexpressed by microglia located in the cerebellum when compared to cortical microglia. Similarly, CD45 or CXCR3 were described to be less expressed in the hippocampus in comparison to the other regions, while TREM2 was differentially expressed between microglia in the cerebellum and cortex (de Haas et al., 2008). Later on, Doorn et al. (2015) investigated baseline differences in microglial expression of genes in brain regions associated with PD, including substantia nigra, striatum, olfactory bulb, hippocampus, or amygdala of rats. The authors did not detect differences in the expression levels of *Aif1*, *Cd11b*, or *Tlr2* genes in microglia isolated from those regions. However, the expression levels of the phagocytic and pro-inflammatory markers *Cd68* and *Il1b* were higher in the olfactory bulb compared to the other brain regions. Besides, *Cd68* expression was higher in the striatum compared to the amygdala and *Tnf* was overexpressed in the substantia nigra (Doorn et al., 2015).

Along with a deeper understanding of microglial heterogeneity across brain regions, the raise of single-cell transcriptomic technologies has been a breakthrough toward further revealing the cellular diversity of the brain at single-cell resolution. In this context, a pioneer work from Tasic et al. (2016) enabled to build a cell taxonomy atlas of the murine cortex identifying up to 49 transcriptomic cell types, 7 of them being non-neuronal cell types, including microglia. In the same year, two key studies helped to understand microglia diversity across brain regions, although not at single-cell resolution yet. More specifically, Grabert et al. (2016) conducted a large RNA-sequencing analysis of isolated microglial cells from the mouse cortex, hippocampus, striatum, and cerebellum. This study demonstrated that the most variable gene ontology terms discriminating the analyzed brain regions were related to metabolism and immune regulation. Besides, microglial transcriptomic heterogeneity clustered into three different signatures: the “cortex, hippocampus, and striatum,” the “cerebellum and hippocampus,” and the “cerebellum” transcriptomes. Importantly, the authors showed that microglia from the cerebellum exhibit a specific “immune-vigilant” transcriptional signature when compared to the others brain regions (Grabert et al., 2016). In addition, Verdonk et al. (2016) expanded the knowledge on regional heterogeneity by shedding light on microglia morphology using an automated method based on 3D reconstruction. With this technique, microglial morphologies have been analyzed according to the complexity of primary ramifications (CI, complexity index) and the total 2D area covered by ramifications (CEA, covered environment area). In line with the transcriptomic findings obtained by Grabert et al. (2016), microglial morphology from the cerebellum was the most diverse. Indeed, cerebellar microglia had smaller CI and CEA in comparison to microglia from the hippocampus, frontal cortex and striatum, which exhibit similar cell body and cellular area (Verdonk et al., 2016). Still in 2016, the first single cell study addressing microglia diversity along development was published by Matcovitch-Natan et al. (2016) who specifically defined early-stage cycling (e.g., *Dab2*, *Mcm5*, *Lyz2)*, synaptic pruning (e.g., *Crybb1*, *Csf1*, *Cxcr2*), and adult immune surveillant (e.g., *MafB*, *Cd14*, *Mef2a*) microglia.

It was in 2017 when De Biase et al. (2017) investigated microglia diversity across different areas of the basal ganglia, including the nucleus accumbens, ventral tegmental area, substantia nigra pars compacta, and substantia nigra pars reticulata, using a combination of morphological and transcriptomic analyses. The authors observed that microglia in the striatum and the nucleus accumbens displayed a higher complexity when compared to the others sub-regions. Further, they reported variability in terms of microglial density within the basal ganglia, detecting a higher number of microglial cells within the substantia nigra pars reticulata when compared with the substantia nigra pars compacta and the ventral tegmental area. Interestingly, they also reported a uniform density of all the other cell types within the basal ganglia, interrogating the origin of the region-specific microglial proliferation capacities. Lastly, they described different microglial transcriptional signatures, especially within the ventral tegmental area, where genes related to mitochondrial function, metabolism, oxidative signaling, or lysosomal function were differentially expressed when compared to the other regions (De Biase et al., 2017).

In 2018, two major studies addressing sexual dichotomy at the transcriptional level have been conducted. In both cases, biological processes related to the immune phenotype (e.g., cytokine production, expression of antigen-presenting cell markers and purinergic receptors) were over-represented in male microglia (Guneykaya et al., 2018; Villa et al., 2018). Further, female microglia have been linked to neuronal processes (e.g., perpetuation of neuronal transmission or promotion of neuroprotective mechanisms), as well as have been described to be more susceptible to microbiota modifications (Villa et al., 2019).

Doubtless, 2019 has been the year when microglial heterogeneity has been mostly elucidated at single-cell resolution. For example, Bottcher et al. (2019), by using mass-cytometry, described nine different human microglial substates across the subventricular zone, thalamus, cerebellum, and the temporal and lateral lobes, with the subventricular zone displaying a more distinct signature compared with the other brain regions. In mice, Hammond et al. (2019), by using single cell technology, found high microglial heterogeneity in young mice (E14.5 and P5), with eight different substates of microglia, for which the expression of markers such as *Arg1*, *Rrm2*, *Hmox1*, or *Spp1* differed. In addition, the authors aimed to identify gender-specific differences of microglia across three different ages (E14.5, P4/P5, and P100), but did not detect main differences between males and females, except for the expression of chromosome-specific genes, such as *Eif2s3y* and *Xist* (Hammond et al., 2019). In addition to the study of Hammond et al. (2019), Li et al. (2019) described the so-called proliferative-region-associated microglia (PAM), a specific microglial subtype located in the developing cerebellar white matter and corpus callosum, which is characterized by the expression of *Spp1* and *Gpnmb*. Lastly, Masuda et al. (2019) conducted a more detailed single-cell study, mainly focused on microglia, in which their heterogeneity has been addressed across specific brain regions, such as the corpus callosum, cerebellum, cortex, hippocampus, and facial nucleus at different mouse ages. The authors identified 10 main substates during development with differences in the expression of microglial markers, such as *Tmem119*, *Malat1*, *Lamp1*, or *Apoe* (Masuda et al., 2019). Notably, in the same study, four different substates of microglia have also been described in the cortex of the human brain (Masuda et al., 2019). In parallel, microglial diversity between the gray and white matter has been studied at the transcriptomic level in the human brain (van der Poel et al., 2019). While microglia located in the gray matter were enriched in genes related to cytokine signaling, microglia in the white matter were characterized by genes involved in chemotaxis and inflammatory responses (van der Poel et al., 2019). In the same year, an automated method named MIC-MAC (Microglia and Immune Cells Morphologies Analyzer and Classifier) enabled to evaluate microglial density and morphologies at single-cell level in the hippocampi of human and mouse brains. This method allowed the clustering of microglial subpopulations based on their similarities in a 3D environment and assisted the identification of 10 different microglial subsets within the mouse and the human hippocampus, revealing a unique subset of human microglia (Salamanca et al., 2019). Still regarding microglia diversity in the human brain, Sankowski et al. (2019) recently applied high dimensional techniques and identified up to eight clusters with differential expression of microglia core genes (e.g., *CX3CR1* and *TMEM119*), genes related to major histocompatibility complex II (e.g., *HLA-DRA* and *CD74*) as well as chemokines and cytokines (e.g., *CCL2* and *IL1B*). In the same study, the authors explored regional-associated differences in the temporal lobe between the gray and white matter and detected higher expression levels of immune genes in microglia located in the latter (Sankowski et al., 2019). Later in 2019, Geirsdottir et al. (2019) studied microglia diversity across a various range of species across the evolutionary tree, including mouse and human. The authors used a combination of single cell transcriptomics and 3D reconstruction to study microglia morphology. In this particular study, the authors showed a conserved morphological pattern of parenchymal microglial cells across the species. However, they called attention on variations observed in terms of dendrite length, number of segments, branch points, and terminal points between the cortical and cerebellum microglia in mice and humans. From the transcriptomic point of view, microglia express a set of core genes that are conserved across species, with human microglia displaying a more pronounced heterogeneity than other mammals. Lastly, microglial genes related to the complement pathway, phagocytosis, or genes implicated in neurodegenerative diseases differ between rodents and primates (Geirsdottir et al., 2019).

## Microglia Adaptation and Diversity in Brain Diseases

**FIGURE 3:**
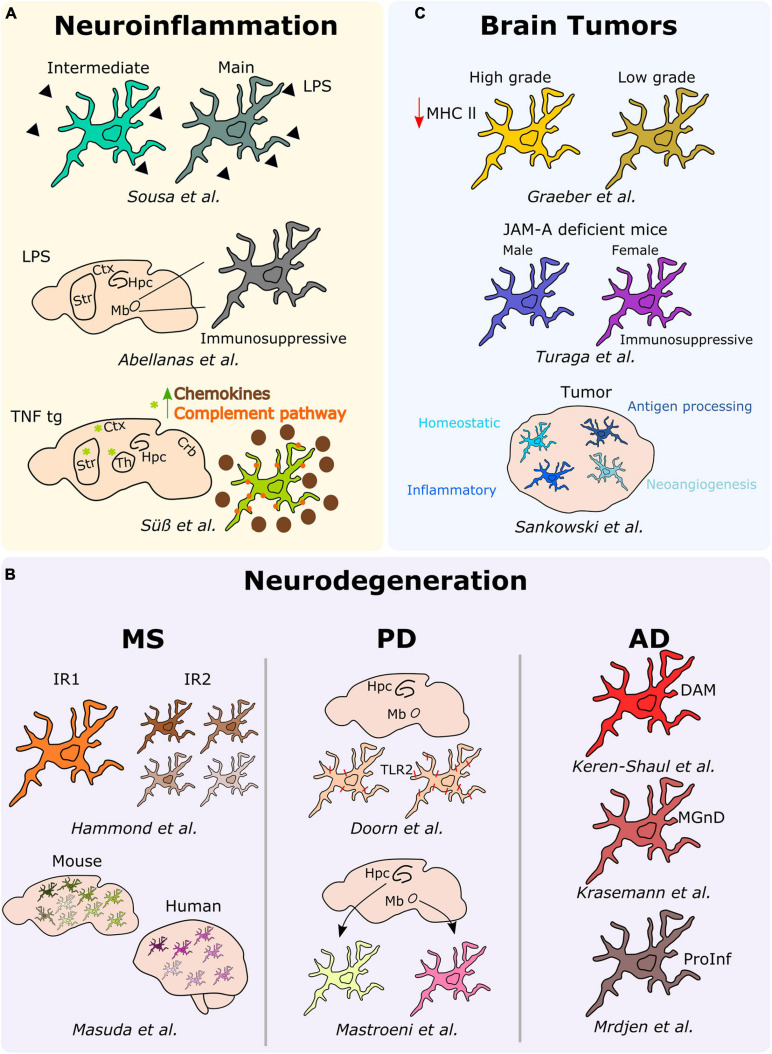
Microglial heterogeneity in different neurological diseases. Schematic description of the main results over the main studies addressing microglia heterogeneity in neuroinflammation, neurodegeneration (MS, PD, and AD), and brain tumors. Hpc, hippocampus; Mb, midbrain; Ctx, cortex; Str, striatum; Th, thalamus; DAM, disease-associated microglia; MGnD, microglial neurodegenerative phenotype; ProInf, pro-inflammatory; IR1, injury responsive 1; IR2, injury responsive 2.

Microglial cells scan the brain parenchyma and react to specific threats to avoid a disturbance of the critical, fine-tuned activities of the CNS. As mentioned previously, the phenotypic analysis of microglia in the healthy brain parenchyma revealed specific poised subsets, which might eventually support or harm the neuronal network under specific vulnerabilities. For example, this is all the more important for the understanding of CNS disorders exhibiting regional-specific and cellular pathological hallmarks, as seen in various neurodegenerative diseases, including AD (entorhinal cortex and hippocampus) and PD (nigrostriatal pathway). Therefore, in this chapter we extend the study of microglial heterogeneity in a disease-associated context. More specifically, we tackle microglia diversity associated with neuroinflammatory and neurodegenerative diseases as well as with brain tumors (Figure 3).

### Neuroinflammatory and Neurodegenerative Diseases

In an attempt to elucidate potential heterogeneous responses of microglia under neuroinflammatory conditions, we analyzed *ex vivo* pre-sorted cells from the mouse brain following a peripheral acute endotoxin challenge, a common model used as a paradigm to study the effect of systemic bacterial infections, ultimately leading or not to neurodegeneration (Bodea et al., 2014). By applying single-cell RNA-sequencing, we demonstrated that the microglial response associated with a peripheral injection of lipopolysaccharide (LPS), most probably induced by a transient serum cytokine storm, rather than stimulated by the response to the TLR ligand that might not reach the brain parenchyma (Banks and Robinson, 2010; Shemer et al., 2020) is heterogeneous. Specifically, we identified two discrete reactive states characterized by various levels of activation and showed that inflammation-induced microglia signatures are distinct from neurodegenerative disease-associated profiles (Sousa et al., 2018). Notably, a similar study conducted a year later showed that microglia isolated from the midbrain of peripherally LPS injected mice adopt an immunosuppressive phenotype in comparison to microglia located within the striatum (Abellanas et al., 2019), thus suggesting that distinct microglia reactions toward neuroinflammatory threats might be region-dependent. In this context, the analysis of microglia phenotypes associated with chronic peripheral inflammation in TNF transgenic mice revealed distinct signatures across different brain regions, including the cortex, striatum, hippocampus, thalamus, and cerebellum. Indeed, microglia located in the cortex, striatum, and thalamus of the transgenic mice clustered together, and their transcriptome significantly differed from the other brain regions. More specifically, microglial cells located within the cortex, striatum and thalamus were characterized by the overexpression of inflammatory genes, such as *Cxcl13*, *Ccl2*, *C3*, and *C4b*, thus suggesting a more pronounced reactive state of microglia under persistent inflammation in these specific regions (Süß et al., 2020).

In the context of neuroinflammatory diseases, multiple sclerosis (MS) is the most common inflammatory, demyelinating and neurodegenerative disorder of the CNS. In this perspective, Hammond et al. (2019) studied microglia diversity in the white matter of mice injected with lysolecithin (LPC), a commonly used mouse model of MS. In this model, microglial cells initially segregated into two clusters, with the so-called injury responsive 1 (IR1) cluster mainly composed by microglial cells of the control group, whereas the IR2 cluster constituted by microglia from LCP injured mice. Further, the authors revealed that IR2 subset was composed by four sub-clusters representing different microglial subtypes or responses in LCP mice differing in the expression levels of proliferative (e.g., *Birc5*) and inflammatory (e.g., *Cxcl10* or *Ccl4*) markers, suggesting that microglia acquire different phenotypes to respond to demyelination (Hammond et al., 2019). Similarly, Masuda et al. (2019) investigated microglial heterogeneity in a MS and neurodegeneration-associated mouse model, respectively the cuprizone and the unilateral facial nerve axotomy models. In this study, the authors describe up to nine different subtypes of microglia displaying differences in the expression levels of inflammatory (e.g., *Spp1*, *Ccl4*, *Cybb*) or MHC-II-related (e.g., *Cd74*, *H2-Aa*, *H2-Ab1*) markers among others (Masuda et al., 2019). Remarkably, a similar microglial heterogeneity has been confirmed in MS patients. More specifically, microglial cells segregated into seven different subtypes, with differential expression levels of chemokines and cytokines (*CCL4* or *ERG2*), MHC-II-related proteins (*CD74* or *HLA-DRA*), and activation markers (*SPP1* or *CTSD*) (Masuda et al., 2019).

In the context of neurodegenerative diseases, Doorn et al. (2014) took advantage of immunohistochemical analyses to examine the expression of TLR2, a known receptor involved in the activation of microglia following its interaction with alpha-synuclein in the substantia nigra and hippocampus of patients with PD and incidental Lewy Body Disease (iLBD), a prodromal state of PD. In iLBD patients, TLR2 microglia expression regionally differed between the substantia nigra and hippocampus. Additionally, Mastroeni et al. (2018) studied the human regional microglial profile associated with PD and AD. More specifically, they conducted RNA-sequencing of isolated microglia from the two most vulnerable brain regions affected in PD and AD, the substantia nigra and the hippocampus CA1, respectively (Mastroeni et al., 2018). They uncovered regional differences highlighting 313 differentially expressed genes between microglia located within the substantia nigra of PD samples and the corresponding cells located in the hippocampus CA1. These differential expressed genes reflected changes in behavior, synaptic transmission or regulation of transport. In the AD samples, 104 differential expressed genes associated with synaptic transmission, cell-cell signaling, or metal ion transport have been detected between microglia located in the hippocampus CA1 and substantia nigra (Mastroeni et al., 2018). In a similar context, Keren-Shaul et al. (2017) used a classical mouse model of AD, the 5XFAD, to study microglial subsets associated with AD at the single-cell level. Notably, the authors depicted a specific population of microglial cells associated with AD, namely disease-associated microglia (DAM). They uncovered that the Trem2 associated pathway, which confers them a higher phagocytic capacity, drives the acquisition of the DAM phenotype. This specific population is supposed to be located around the amyloid-ß plaques and has been confirmed to be also present in the brains of AD patients (Keren-Shaul et al., 2017). Consistently, a transcriptional phenotype of dysfunctional microglia in neurodegenerative diseases, termed “microglial neurodegenerative phenotype” (MGnD), driven by the TREM2-APOE pathway, has been concomitantly described (Krasemann et al., 2017). Further, Mrdjen et al. (2018) took advantage of the CyTOF to identify a microglial subtype in another mouse model of AD, the APP/PS1. Indeed, they identified a subset of microglial cells characterized by the overexpression of phagocytic (e.g., *Cd11c* and *Cd14*), activation (e.g., *Cd86* and *Cd44*) as well as MHC-II-associated markers. In this subset, in line with a pro-inflammatory phenotype, the expression levels of the homeostatic microglia markers (e.g., *Cx3cr1* or *Siglec-H*) were downregulated (Mrdjen et al., 2018).

### Brain Tumors

In the past, inter-tumor microglial morphological heterogeneity has been described, for example, across different gliomas, where microglia display a more pronounced amoeboid morphology in high-grade tumors, while they are ramified in low-grade tumors. These differences were also associated with lower levels of MHC-II expression in high-grade gliomas when compared to their low-grade counterparts (Graeber et al., 2002). Interestingly, microglia diversity associated with brain tumors, and specifically in GBM, has been investigated also taking into account gender specificities. Turaga et al. (2020), while conducting a study regarding the implication of junctional adhesion molecule-A (JAM-A), noticed a poorer prognosis in GBM implanted female JAM-A deficient mice when compared to the corresponding implanted males. Notably, the authors reported an upregulation of the anti-inflammatory genes *Fizz1* and *Ifi202b* in microglia from female JAM-A deficient mice compared to their male counterparts (Turaga et al., 2020). Sankowski et al. (2019), by combining scRNA-seq and CyTOF analyses on human brain samples, including samples obtained from GBM patients, discovered various subpopulations of microglial cells within the tumor. Indeed, they defined a continuum from control-enriched clusters to glioma-associated microglial clusters, the latest being characterized by a decreased expression of core microglial signature genes (*CX3CR1*, *SELPLG*, *P2RY12*, *CSFR1*) and an increased expression of metabolic, inflammatory and interferon-associated genes (*CD163*, *APOE*, *LPL*, *IFI27*, *IFI44*, *SPP1*). Interestingly, the in-between clusters were exhibiting differential expression levels of the previous cited genes, but also hypoxia-related (*VEGFA*, *HIF1A*) and antigen processing MHCI-related genes. Additionally, CyTOF analyses enabled the detection of differential proportions of HLA-DR, TREM2, APOE, and GPR56, confirming major differences between control and glioma-associated microglia also at the protein level (Sankowski et al., 2019). Although only poorly investigated to date, also secondary brain metastases can alter microglial properties, or—vice versa—microglia may even pave the way for an enhanced cerebral dissemination of peripheral tumor cells via increased secretion of IGF-1 and CCL20 together with a reduced expression of SIRP-alpha, the latter leading to impaired phagocytic properties (Simon et al., 2020; Wu et al., 2020). Very recently, the first experimental studies addressing the composition of primary and secondary myeloid cell populations at single-cell level have been performed in mouse models indicating that the genetic programming of brain metastasis-associated myeloid cells is a very early and stable event (Schulz et al., 2020).

## Conclusion and Perspectives

Undoubtedly, the ability to acquire different resting and activated phenotypes confers microglia the advantage to be a plastic and adaptive cell type in the CNS. Along with this review, we have highlighted a large number of studies demonstrating that microglia are far from being a resting or homogenous cell population. For example, in the healthy mouse brain, microglia heterogeneity across various regions has been described in terms of density, morphology, molecular signatures, and metabolism (Tan et al., 2020). For translational purposes, it will be critical to consider that microglia heterogeneity has been suggested to be even higher in the human brain compared to mouse (Prinz et al., 2019). Notably, regarding neurodegenerative diseases little is known about microglial heterogeneity in PD. Hence, future efforts will need to be directed at understanding if specific microglia subsets might differently contribute to PD pathology. In the context of brain tumors, with the difficulty to define reliable markers deciphering macrophages and microglia, the majority of the transcriptomic studies have been focusing on defining a glioma-associated microglial signature in different models, but not assessing their heterogeneity within the tumor mass (Darmanis et al., 2017; Walentynowicz et al., 2018; Maas et al., 2020). The study by Sankowski et al. (2019), being the first one highlighting specific heterogeneous signatures of microglial cells in GBM, calls attention on the lack of knowledge on microglial heterogeneity in that context. Hence, further studies would need to be directed at understanding the implication of different microglial subsets in glioma development and progression (Sankowski et al., 2019). Similarly detrimental, however, much more frequently than primary brain tumors, also brain metastases, at least experimentally, show a relevant contribution of microglia in the establishment and progression of secondary brain tumors, therefore constituting a target for future treatment strategies.

Taken together, microglial variety embraces fundamental aspects, such as spatial-temporal organization, which is present in the healthy and diseased brain. Thanks to the development of high-throughput technologies, including single-cell approaches, different microglial subsets have been unraveled, indicating that microglia are able to adapt to specific environments across particular niches in the healthy brain. In addition, single-cell analyses have been conducted to study microglia associated with inflammation and neurological disorders untangling specific subsets of cells that might differently contribute to each specific pathology (Masuda et al., 2020). Notwithstanding, a crucial aspect that would need to be tackled in future studies would be to understand the functional implication of specific microglial subpopulations across particular neurological diseases, which might enable to explore novel avenues to target neuroinflammation and microglial cells in a specialized context-dependent manner.

## Author Contributions

OUH and AM conceived the manuscript. OUH, LR, and AM wrote the manuscript. OUH created the figures. OUH, LR, MM, and AM critically revised and approved the final version of the manuscript. All authors contributed to the article and approved the submitted version.

## Conflict of Interest

The authors declare that the research was conducted in the absence of any commercial or financial relationships that could be construed as a potential conflict of interest.
